# Elevated Troponin, D-Dimers and D-Dimers/Fibrinogen Ratio Increase Mortality Risk in Remdesivir-Treated COVID-19 Patients

**DOI:** 10.3390/jpm15110519

**Published:** 2025-10-31

**Authors:** Georgia Ragia, Gavriela Voulgaridou, Vasileios Tsirozis, Triada Exiara, Vangelis G. Manolopoulos

**Affiliations:** 1 Laboratory of Pharmacology, Medical School, Democritus University of Thrace, Dragana Campus, 68100 Alexandroupolis, Greece; gragia@pharm.auth.gr (G.R.); gabivoulg@gmail.com (G.V.); 2 Individualised Medicine & Pharmacological Research Solutions (IMPReS) Center, Dragana Campus, 68100 Alexandroupolis, Greece; 3 Department of Internal Medicine, General Hospital of Komotini “Sismanogleio”, 69133 Rodopi, Greece; vtsirozis4@gmail.com (V.T.); texiara@otenet.gr (T.E.); 4 Clinical Pharmacology Unit, Academic General Hospital of Alexandroupolis, Dragana Campus, 68100 Alexandroupolis, Greece

**Keywords:** COVID-19, biomarkers, mortality risk factors, remdesivir, troponin, D-dimers, fibrinogen

## Abstract

**Background/Objectives**: Identification, monitoring, and modulation of biochemical markers that increase risk of severe illness and death from COVID-19 are crucial for effective disease treatment. This study aims to investigate the prognostic value of baseline troponin, D-dimers, fibrinogen, and D-dimers/fibrinogen ratio (DFR) as biomarkers for mortality in remdesivir-treated patients, and additionally, to investigate the impact of remdesivir treatment on troponin, D-dimers, fibrinogen, and DFR levels during disease course. **Methods**: We retrospectively analyzed the demographic, biochemical, and clinical characteristics of 549 (47.5% male, mean age 69.6 ± 14.7) hospitalized COVID-19 patients, all treated with remdesivir. **Results**: A total of 106 patients (19.3%) died during hospitalization. Elevated baseline troponin levels and D-dimers increased risk of death (HR 2.374, 95% CI 1.343–4.197; *p* = 0.003, adjusted for comorbidities and HR 1.862, 95% CI 1.127–3.076, *p* = 0.015, unadjusted, respectively). After remdesivir treatment, death risk was increased by elevated troponin (HR 2.010, 95% CI 1.219–3.316, *p* = 0.006), D-dimers (HR 2.207, 95% CI 1.254–3.882, *p* = 0.006) and DFR (HR, 3.816, 95% CI 1.567–9.291, *p* = 0.003) levels, in models adjusted for age, sex, and comorbidities. Remdesivir treatment decreased fibrinogen levels both in survivors (*p* < 0.001) and non-survivors (*p* < 0.001). In survivors, remdesivir treatment significantly decreased troponin levels (*p* < 0.001) and D-dimers (*p* < 0.001), whereas in non-survivors, it increased DFR (*p* < 0.001) and D-dimers (*p* < 0.001). **Conclusions**: In addition to its antiviral action, remdesivir treatment was associated with changes in coagulation biomarkers, potentially preventing patients from the COVID-19-provoked hypercoagulable state. Troponin, D-dimers, and DFR hold a critical role in predicting mortality risk among COVID-19 patients treated with remdesivir and can aid in identifying individuals at increased risk of death.

## 1. Introduction

Coronavirus disease (COVID-19) drug treatment is still a medical challenge and the pursuit of effective therapeutic interventions and reliable prognostic factors for COVID-19 remains of paramount importance. Several virus- and host-targeted agents have been used in COVID-19 after being found effective in SARS-Cov-2 suppression in pre-clinical and clinical studies [1]. Nevertheless, to date, no specific pharmaceutical agent is yet universally recommended for the treatment of COVID-19. Treatment guidelines for COVID-19, developed by the European Society of Clinical Microbiology and Infectious Diseases (ESCMID), advocate for tailored therapeutic regimens contingent upon severity and the clinical condition of the patient. More specifically, ESCMID recommends systemic corticosteroids or tocilizumab for critically ill COVID-19 patients or patients with severe cases, remdesivir for those requiring hospitalization, and casirivimab and imdevimab for high-risk patients with mild-to-moderate COVID-19 [2].

Remdesivir is a nucleoside prodrug, exerting its antiviral effects via its active triphosphate analog through inhibition of viral RNA-dependent RNA polymerases [3]. These enzymes exhibit structural conservation and play a pivotal role in the replication of various viruses, including SARS-CoV-2 [3]. Notably, remdesivir decreased early-stage mortality and diminished the need for high-flow oxygen supplementation and invasive mechanical ventilation in COVID-19 patients admitted to hospitals [4,5]. Other randomized clinical trials have additionally demonstrated that hospitalized patients treated with remdesivir experienced a shorter time to recovery, alongside a reduction in the number of days that patients required mechanical ventilation [6] or had lower risk of hospitalization or death [7,8] compared to those receiving placebo.

Biomarker discovery for COVID-19 progression and outcome has also been a challenge. The early observation that COVID-19 provoked thrombo-inflammation and increased incidence of venous thromboembolism (VTE) and pulmonary embolism, pointed out coagulation biomarkers, namely fibrinogen, D-dimers, and D-dimers/fibrinogen ratio (DFR) as attractive candidates to predict COVID-19-associated thrombogenicity and related mortality [9,10]. Notably, elevated levels of coagulation markers, specifically of D-dimers and fibrinogen, have been suggested to be significant determinants of prognosis in COVID-19 patients [11]. Additionally, DFR, compared to D-dimer and fibrinogen alone, has the potential for improved diagnostic accuracy and specificity in detecting thromboembolic events, differentiating cases presenting elevated D-dimer from other physiological processes [12].

COVID-19 has been associated with life-threatening or fatal cardiovascular manifestations, such as sudden heart failure, arrhythmia, and cardiac arrest [13]. Troponin has also emerged as a potential prognostic biomarker of myocardial injury and increased mortality in COVID-19 patients [14,15,16]. Elevated high-sensitivity cardiac troponin T levels in COVID-19 patients suggested COVID-19 induced cardiac injury, and was linked to increased risk of severe COVID-19, leading to patient mechanical ventilation and admission to the intensive care unit (ICU) [17,18]. Moreover, D-dimer may account for a substantial proportion of troponin variability [19]; thus, combining troponin with D-dimer has emerged as a triage and prognosis biomarker that may provide better specificity for cardiovascular outcomes and COVID-19 mortality [20], especially when symptoms of life-threatening conditions overlap [21,22].

The potential prognostic value, however, of troponin, fibrinogen, D-dimers, and DFR in the COVID-19 course in remdesivir-treated patients is scarcely studied. Currently, only two studies have investigated the potential association of D-dimers with COVID-19 mortality in remdesivir-treated patients [8,23]. We herein examine the prognostic significance of troponin, D-dimers, fibrinogen, and DFR for COVID-19 mortality, both independently and in association with remdesivir treatment. Specifically, in the present study, we have assessed (a) the predictive value for mortality of baseline (upon admission to hospital and prior to remdesivir treatment) troponin, fibrinogen, D-dimers, and DFR in COVID-19 hospitalized patients, (b) their impact on remdesivir treatment, and (c) whether remdesivir administration alters troponin, fibrinogen, D-dimers, and DFR levels, thus, impacting mortality.

## 2. Materials and Methods

### 2.1. Study Design and Study Population

In our study, we have retrieved data for the total population of patients with COVID-19 who were admitted to the General Hospital of Komotini “Sismanogleio”, Rodopi, Greece, and received remdesivir treatment from November 2020 to December 2022. The hospital served as the reference remdesivir treatment hospital in the Rodopi region for COVID-19. In the study population, remdesivir was the sole antiviral drug administered. Specifically, a total of 549 consecutive patients were retrospectively included in the study. SARS-CoV-2 was confirmed in all patients by real-time polymerase chain reaction (PCR). Age <18 years was the only exclusion criterion. The protocol of the study was approved by the institutional ethics review board of General Greek Hospital of Komotini “Sismanogleio” (100/40/19-4-2022) and was conducted in accordance with the Helsinki Declaration.

### 2.2. Remdesivir Treatment

Remdesivir (Veklury, Gilead Sciences) was dissolved in 250 mL of 0.9% sodium chloride and was administered intravenously during a five-day course (200 mg on the first day and 100 mg for the next four days). Administration lasted >120 min.

### 2.3. Data Collection

Data were extracted from medical records, and included demographic characteristics (age and sex), and medical history (pulmonary disease such as asthma, chronic obstructive pulmonary disease, pulmonary embolism, hypertension, cardiovascular diseases (CVDs) such as ischemic stroke, coronary artery disease, atrial fibrillation, and other arrhythmias, heart failure, and atherosclerosis, type 2 diabetes mellitus, obesity, and cancer). Patient clinical data, such as remdesivir dosage and vaccination before COVID-19 disease, as well as clinical course of COVID-19 disease (length of hospitalization, intubation, death) and COVID-19 symptoms (e.g., pain, fever, nausea, and dyspnea) were recorded. Biochemical data measurements included troponin, fibrinogen, and D-dimers upon admission to the hospital and prior to remdesivir treatment, and after remdesivir treatment. DFR was calculated using the formula as described in [12]:

$$DFR=\frac{D-dimers\;(\mu g/mL)}{Fibrinogen\;(mg/dL)}\times 100$$

**Figure 1:**
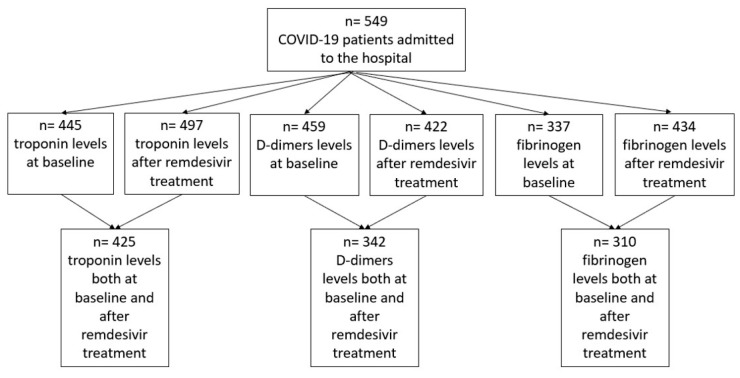
Patient flowchart on troponin, D-dimers, and fibrinogen measurements.

An analytic description of biochemical measurements and the number of patients included in each analysis is provided in Figure 1.

### 2.4. Statistical Analysis

Kolmogorov–Smirnov test was performed to estimate the normality of the distribution of the continuous variables. All continuous variables that have non-parametric distribution are presented as median and interquartile range (IQR). Wilcoxon test was performed to compare the biochemical measurements before and after remdesivir uptake. Mann–Whitney test was performed to compare age, total dose of remdesivir, length of hospitalization, and biochemical measurements in stratified analyses by sex or by surviving. Categorical variables are presented in absolute values and percentages. Chi-square test was used to compare frequency of intubation, vaccination status, mortality, and comorbidities between men and women.

Receiver–operator characteristic (ROC) analysis was performed to determine the mortality predictive value of troponin, fibrinogen, and D-dimers levels, both before and after remdesivir treatment, and the optimal cut-off values, sensitivity and specificity were determined. Survival curves (Kaplan–Meier) were used to compare the probability of survival over time in remdesivir-treated patients. Analyses of Cox proportional hazard models were utilized to evaluate the prognostic significance of the biomarkers and clinical variables. Statistical Package for the Social Sciences (SPSS) version 27.0 for windows (IBM Corp., Armonk, NY, USA) and packages “pROC” and “dplyr” in R software were used for all statistical analyses. The statistical significance was defined as *p* < 0.05 for all analyses.

## 3. Results

### 3.1. Clinical and Laboratory Characteristics of the Study Population

The total demographic, clinical, and biochemical characteristics of patient population, as well as stratified by mortality, are described in Table 1. Median age was 72 years (IQR 59–81) and 47.5% of patients were male. Out of 549 patients, 86.5% had underlying diseases; hypertension was the most common comorbidity (65.6%), followed by CVDs (35.2%). The most frequent COVID-19 symptoms were cough (64.7%), fever (62.3%), and dyspnea (48.8%).

One hundred and six (19.3%) patients died during hospitalization (non-survivor group). As expected, patients who died had prolonged hospitalization compared to survivors (12 vs. 8 median days *p* < 0.001). Significant differences were present in age (75 vs. 77.5 median years, *p* < 0.001), intubation (2% vs. 32%, *p* < 0.001), and prevalence of CVDs (31.6 vs. 49.1%, *p* = 0.006) between survivors and non-survivors. Interestingly, COVID-19 survivors had relatively more severe COVID-19 clinical symptoms (fever; 64.3 vs. 53.8%, *p* = 0.006, cough; 67.5 vs. 52.8%, *p* < 0.001) compared to non-survivors (Table 1).

Between the two groups (survivors vs. non-survivors), baseline troponin levels (levels at admission) were higher in non-survivors (*p* < 0.001); however, no significant difference was found between the two groups after remdesivir treatment (*p* = 0.201). In addition, baseline D-dimers levels (*p* = 0.004) and DFR (*p* < 0.001) were increased in non-survivors and remained increased after remdesivir treatment (*p* = 0.003 and *p* < 0.001, respectively). Increased fibrinogen levels were observed in survivors after remdesivir treatment (*p* < 0.001).

### 3.2. Prognostic Mortality Accuracy and Survival Analysis of Troponin, D-Dimers, Fibrinogen, and DFR

**Figure 2:**
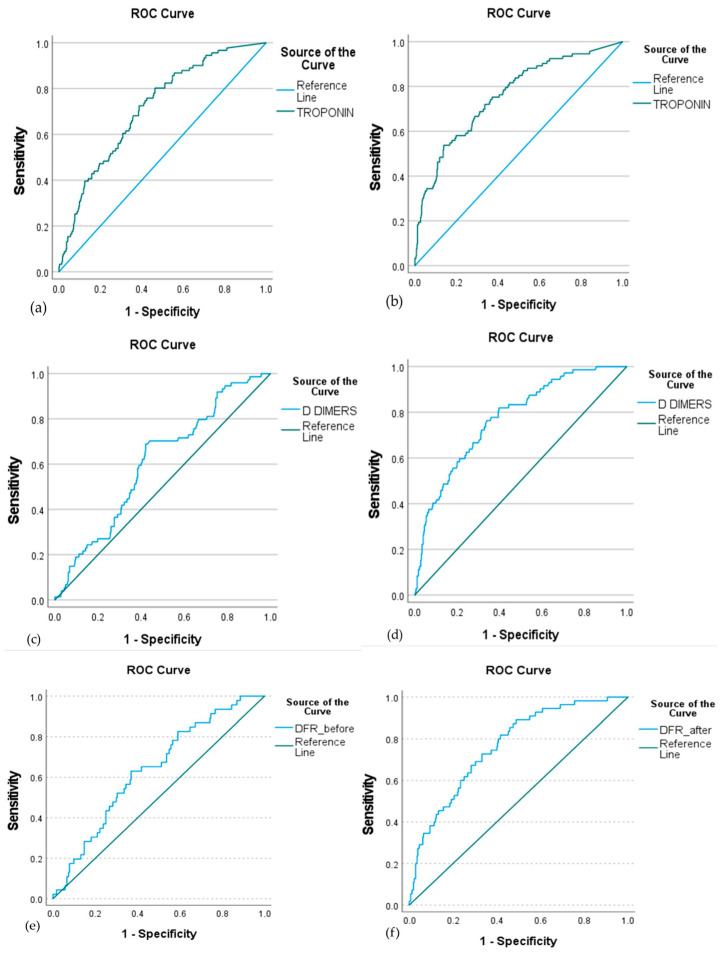
ROC curve of troponin, D-dimers, and DFR at baseline (**a**,**c**,**e**, respectively) and after remdesivir treatment (**b**,**d**,**f**, respectively) to predict mortality in COVID-19 patients. The ROC analysis demonstrated significant discriminatory ability for all three biomarkers both before and after treatment. Baseline AUC values were 0.715 for troponin, 0.606 for D-dimers, and 0.638 for DFR, while post-treatment AUCs were 0.759, 0.773, and 0.765, respectively. These results indicate that higher levels of troponin, D-dimers, and DFR are associated with increased mortality risk, suggesting their prognostic utility in remdesivir-treated patients.

In ROC curve analysis (Figure 2, Table 2), troponin, D-dimers, and DFR showed significant diagnostic accuracy in determining mortality risk both at baseline (AUC: 0.715, 0.606, and 0.638, respectively) and after remdesivir treatment (AUC: 0.759, 0.773, and 0.765, respectively). Fibrinogen levels did not show prognostic accuracy for mortality, neither before nor after remdesivir treatment .

**Figure 3:**
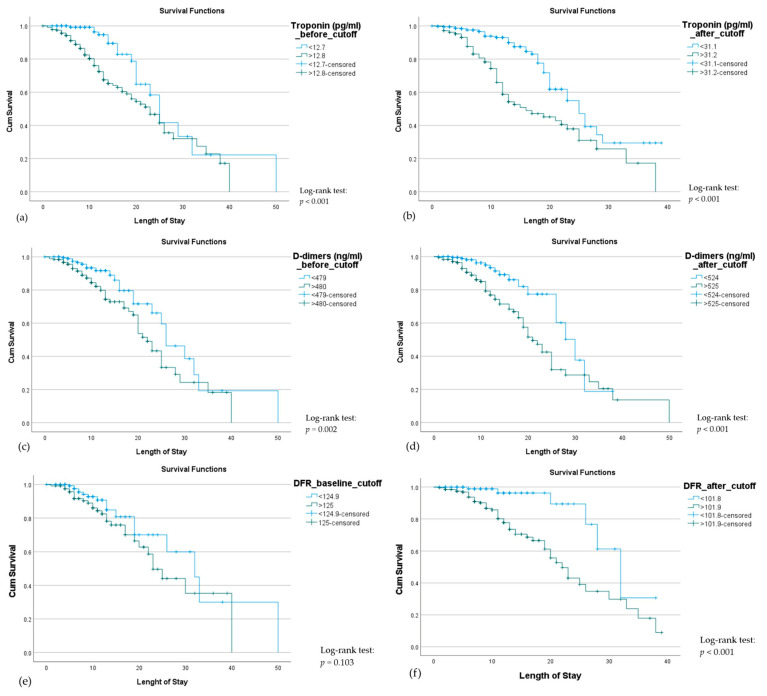
Kaplan–Meier curves illustrating mortality among hospitalized COVID-19 patients according to troponin, D-dimers, and DFR levels at baseline (**a**,**c**,**e**) and after (**b**,**d**,**f**) remdesivir treatment. Patients with elevated troponin (>12.8 pg/mL baseline, >31.2 pg/mL post-treatment), D-dimers (>480 ng/mL baseline, >525 ng/mL post-treatment), or DFR (>0.101 post-treatment) had significantly lower survival (log-rank *p* < 0.05), while baseline DFR was not significant (*p* = 0.103).

Kaplan–Meier curves for troponin, D-dimers, and DFR at baseline and after remdesivir administration are shown in Figure 3. Troponin level >12.8 pg/mL at baseline (*p* < 0.001) and >31.2 pg/mL after remdesivir treatment (*p* < 0.001), and D-dimers level >480 ng/mL at baseline (*p* = 0.002) and >525 ng/mL after remdesivir treatment (*p* < 0.001) were significant predictors of mortality. DFR >0.101 after drug treatment was also a significant predictor of mortality. Therefore, monitoring for troponin, D-dimers, and DFR level increases during remdesivir treatment could be used to monitor therapeutic response.

### 3.3. Cox Regression Models for Mortality in COVID-19 Patients

For each biomarker, both at baseline and after remdesivir treatment, four models were developed to observe the effect of each marker on death in COVID-19 patients. Model 1 is the unadjusted model for each biomarker; model 2 includes the biomarker with additional covariates: age and sex; model 3 includes the biomarker with additional covariates: pulmonary disease, CVDs, hypertension, carcinoma, obesity, and type 2 diabetes mellitus; whereas model 4 includes biomarkers and the combination of covariates of model 2 and model 3. Cut-off values for each biomarker derived from ROC analysis as described earlier.

Both before and after remdesivir treatment, higher troponin levels increased death risk in the different models that were applied (Table 3 and Table 4).

Similar findings associating increased D-dimers levels at admission and after remdesivir treatment with risk of death have derived (Table 5 and Table 6).

Both in unadjusted and adjusted Cox regression models, DFR during admission was not associated with mortality (Table 7). After remdesivir treatment; however, in all Cox regression models, unadjusted and adjusted, high DFR (≥0.101) increased death risk (Table 8).

### 3.4. Remdesivir Effect on Troponin, D-Dimers, Fibrinogen Levels, and DFR in Patients Based on the Outcome of Death

**Figure 4:**
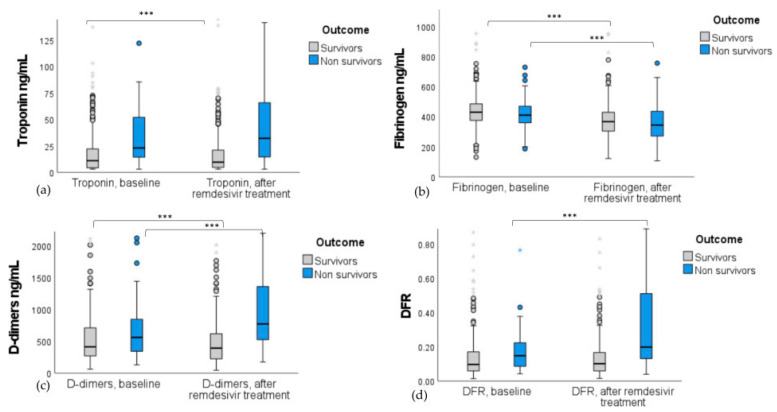
Boxplots with interquartile range of (**a**) troponin, (**b**) fibrinogen, (**c**) D-dimers, and (**d**) DFR differences in remdesivir-treated patients stratified by the outcome of death. Biomarker levels are presented at baseline and after remdesivir administration. Remdesivir significantly reduced troponin and D-dimer levels in survivors (*p* < 0.001) but increased D-dimers and DFR in non-survivors (*p* < 0.001). Fibrinogen levels decreased in both groups (*p* < 0.001). *** indicates significance at *p* < 0.001; Wilcoxon test was used.

Remdesivir decreased troponin (*p* < 0.001; Figure 4a) and D-dimer levels (*p* < 0.001; Figure 4d) in patients who survived and increased DFR levels (*p* < 0.001; Figure 4d) in patients who died. Fibrinogen levels were decreased in both groups (*p* < 0.001 for survivors and non-survivors; Figure 4b), while D-dimers levels increased significantly (*p* < 0.001) in non-survivors (Figure 4c).

## 4. Discussion

In this retrospective cohort study, we aimed to evaluate the prognostic value for mortality of troponin, and coagulation biomarkers fibrinogen, d-dimers, and DFR in hospitalized patients with COVID-19, both at the time of admission and following treatment with remdesivir. We have found that increased troponin and D-dimer levels at baseline or persisting increased levels during remdesivir treatment were associated with increased death risk and, thus, worse COVID-19 prognosis, despite remdesivir treatment. Additionally, we have found that remdesivir impacted levels of fibrinogen and D-dimers in all patients, whereas its effect on troponin levels was noticed only in survivors. Remdesivir only had an effect on DFR in patients who died.

Development of vaccines against SARS-CoV-2 and of therapeutic treatments eventually led to a decrease in COVID-19 death rate. Despite treatment with antiviral drugs, such as remdesivir, mortality rate ranges from 11 to 28% [24,25]. In our study, mortality rate among the 549 hospitalized patients receiving remdesivir was 19.3%. Consistent with other studies, older age [23,26] and CVDs [27,28] were significantly associated with mortality risk despite remdesivir treatment [25]. Interestingly, COVID-19 survivors suffered from more severe COVID-19 symptoms, such as fever and cough; however, these clinical symptoms have been described as predictors of poor prognosis [26]. The use of body temperature as a prognostic indicator in COVID-19 is still under investigation. Published papers debate the relationship of fever with worse clinical outcomes [27], suggesting that early fever, compared to fever per se, may compromise clinical outcomes in COVID-19 patients [28]. Similarly for cough, though it is the most common initial symptom in COVID-19 patients [29], several reports argue its independent correlation with COVID-19 prognosis or survival [30].

In clinical practice, biomarker discovery for COVID-19 death prognosis has been a challenge. We have herein found that the vast majority of patients who died had, at admission, elevated baseline levels of troponin, D-dimers, and DFR compared to survivors. Τroponin exhibited the highest predictive accuracy for death prior to remdesivir treatment. After remdesivir treatment, non-survivors had decreased fibrinogen levels and a sustained increase in D-dimers and DFR compared to survivors. Following remdesivir administration, D-dimers and DFR surpassed troponin in terms of predictive accuracy for mortality.

Traditionally, troponin has served as a robust marker of myocardial injury, including conditions such as coronary artery disease and myocarditis, owing to its exceptional specificity and sensitivity in myocardial injuries diagnosis [31]. Thus, troponin has emerged as a valuable early indicator of the extent of cardiac damage in patients [32]. Elevated troponin levels have also been documented in other clinical conditions, including pulmonary embolism and pulmonary failure [33,34]. The predictive value of troponin for disease severity and death in untreated COVID-19 patients has been previously investigated [33,34,35,36,37,38,39]. Consistent with our findings, increased baseline or peak troponin levels are associated with COVID-19 mortality. In our study, we additionally show that after remdesivir treatment, the prognostic accuracy of troponin levers is even higher in ROC analysis, even after adjusting for age, sex, and underlying diseases. Notably, troponin levels have been observed to rise in hospitalized patients with COVID-19 after remdesivir treatment [40], potentially explaining the heightened risk of cardiac-adverse events in COVID-19 patients treated with remdesivir compared to those administered hydroxychloroquine and azithromycin [41]. Therefore, our findings suggest that the association of troponin with COVID-19 mortality is a prognostic marker irrespective of treatment and it can thus be proposed that both early troponin monitoring and in-treatment monitoring can have a pivotal role in predicting the mortality risk in COVID-19 patients.

Coagulation abnormalities are a common manifestation in hospitalized patients with COVID-19, accompanied by elevated fibrinogen and D-dimer levels [42]; changes in fibrinogen and D-dimers, as a result of inflammation and coagulopathy, suggest coagulation biomarkers as potential predictive factors for mortality in COVID-19 [43]. D-dimers serve as a marker of increased thrombotic activity, reflecting the dynamic balance between fibrin formation and degradation, thereby acting as indicators of coagulation activation and fibrinolysis [44]. Several reports have confirmed the presence of both micro- and macro-thrombi in COVID-19 patients [45] supporting the evaluation of coagulation markers in individuals with COVID-19. Our results indicate that high D-dimer levels at the time of admission among COVID-19 patients increase the risk of mortality. This finding is in line with results already published [37,38,46]. A systematic review with meta-analysis revealed a correlation between elevated D-dimer levels upon admission and increased COVID-19 mortality [47]. Increased D-dimer levels potentially serve as a risk factor of thrombotic events and pulmonary disease [47,48,49]. In clinical practice, D-dimers were associated with the progression of and mortality risk of COVID-19 [50]. Our results also show that elevated D-dimer levels following remdesivir treatment were significantly associated with mortality, even after adjusting for age, sex, and comorbidities. In alignment with our findings, a similar trend of D-dimers with COVID-19 mortality has been reported in very elderly patients treated with remdesivir [8]. Thus, the evaluation of D-dimers can be used for the determination of appropriate treatment strategies; nevertheless, due to limited available data, more research is needed to draw firm conclusions.

For fibrinogen, no differences were found at admission between survivors and non-survivors, and, thus, it did not emerge as a potential prognostic marker. These results are in line with the sole published study by Tang et al., who compared fibrinogen at admission between survivors and non-survivors [46]. However, in other studies, an association has been reported between elevated fibrinogen levels upon admission and COVID-19 adverse outcomes, including disease severity [51,52] and risk of thromboembolic and pulmonary embolism events [51]. This discrepancy can be attributed to the role of blood coagulation on thromboembolic events and inflammation [53,54]. Pro-inflammatory cytokines lead to upregulation of tissue factor (TF) expression, promoting further procoagulant activity [55]. During COVID-19 infection, marked inflammation is evident, indicated by elevated inflammatory markers, such as interleukin-6 (IL-6) and C-reactive protein (CRP) [56,57]. Sui et al. demonstrated a positive correlation between increased inflammatory markers and elevated fibrinogen levels [56]. Consequently, fibrinogen may be used as a promising marker for detecting inflammation in COVID-19 patients upon admission, serving as a potential tool for predicting disease severity rather than death prognosis.

We have also found that an increased DFR stratifies the risk of mortality following remdesivir treatment, though its utility in predicting mortality at admission is limited. It is noteworthy that only Murat et al. have assessed the prognostic value of DFR in COVID-19 patients with a diagnosis of heart failure. The authors have shown that in these patients, DFR was a prognostic marker in hospitalized COVID-19 patients even without treatment [12]. It can thus be suggested that the prognostic value of DFR may be restricted in patients with a background of certain diseases.

We have additionally evaluated the remdesivir effect on troponin and coagulation biomarkers, comparing levels at admission and after remdesivir treatment. Subgroup analyses investigating changes in biomarker levels after treatment, conducted separately for the survivor and non-survivor groups, provide valuable insights for remdesivir biochemical mechanism on COVID-19 progression. Notably, troponin levels exhibited an increase in the survivor group, while non-survivors presented with significantly elevated troponin levels upon admission. This implies that non-survivors may already have incurred substantial cardiac damage prior to treatment, rendering troponin less informative in such cases. Furthermore, our findings indicate a reduction in fibrinogen levels in both the survivor and non-survivor groups, supporting the hypothesis that remdesivir treatment mitigates the inflammatory response and modulates coagulation cascade, potentially preventing the hypercoagulable state provoked by COVID-19 [58]. Conversely, remdesivir was associated with a decrease in D-dimer levels in the survivor group, but in non-survivors, D-dimers remained elevated. Previous reports have highlighted that remdesivir administration in patients with pre-existing CVDs can potentially induce cardiotoxic and proarrhythmic effects, as well as arrhythmias and/or cardiac arrest, especially when co-administered with other medications [41,59]. These observations could explain our finding that remdesivir was associated with an increase in DFR among non-survivors.

The results presented herein were generated from remdesivir-treated patients hospitalized from November 2020 to December 2022. It should be acknowledged that today, COVID-19 has a different course than the years evaluated in the study. According to the European Centre for Disease Prevention and Control, the annotated SARS-CoV-2 variants of interest and under monitoring are Omicron BA.2.86 and Omicron NB.1.8.1 and XFG, respectively. These variants are reported as less likely to cause severe illness compared to previous Omicron sub-variants [60,61,62]. However, some people are still being hospitalized and dying, especially those of older age, whereas there is additionally no guarantee that more severe variants will not emerge in the future. Remdesivir retains antiviral activity against the Omicron variants [63]; therefore, biomarkers that predict the response to approved treatments are currently relevant and, additionally, can serve as a base for any future COVID-19 survival biomarker identification.

Our study focuses on the effect of remdesivir on coagulation factors that could stratify survivors versus non-survivors. Beyond remdesivir, thromboprophylaxis with heparin was also considered in hospitalized COVID-19 patients to reduce mortality. Though for the study cohort, no data is available for heparin use, it is worth mentioning that elevated D-dimer levels have been reported as a factor influencing heparin dose [64]. Additionally, trials have shown that, irrespective of COVID-19, patients with elevated troponin benefit from an antithrombotic strategy including treatment with heparin [65]. Taking all that data together, remdesivir biomarkers or response may be further assessed for their use in the choice and/or therapeutic dose of heparin. In this context, other coagulation markers, such as the activated partial thromboplastin time (aPTT), and the prothrombin time (PT) could be further evaluated as for their prognostic values in COVID-19 thromboprophylaxis response.

Our study has several strengths. The study hospital served as the reference remdesivir treatment hospital in the Rodopi region for COVID-19. Therefore, patient population entirely consists of COVID-19 patients, hospitalized from November 2020 to December 2022, who received remdesivir. To the best of our knowledge, this is the first study that evaluated the effect of troponin and coagulation markers on COVID-19 mortality, both at admission, and after remdesivir treatment. Therefore, our results describe the baseline prognostic value of these biomarkers, and, additionally, their change over remdesivir treatment. We should also acknowledge that unavoidable limitations exist in the study design. Due to the non-uniform ordering of biochemical analyses in real clinical settings, for several patients, data is missing at both timepoints (baseline and after treatment). As a retrospective observational study conducted in a single institution, there is inherent risk of selection bias and limited generalizability to other regions or healthcare settings. A comparable group of patients who did not receive remdesivir or received alternative therapy could not be included in the study. Other factors such as corticosteroid use, tocilizumab administration, vaccination status details, and antiviral timing relative to symptom onset were not included in the models. Finally, details on the severity of COVID-19 were not provided.

## 5. Conclusions

In conclusion, our study shows that troponin, D-dimers, and DFR are predictors for mortality in COVID-19 patients, regardless of whether they have been treated or not with remdesivir. Remdesivir treatment had different effects on these biomarkers, while it uniformly reduces fibrinogen both in survivors and non-survivors. Due to the high prevalence of CVDs in COVID-19 patients, future studies should focus on the clinical utility of screening and monitoring the levels of these biomarkers along with other clinical parameters during the patient’s admission to hospital to reduce the risk of COVID-19 mortality. Towards this direction, prospective validation of findings in multi-center studies with standardized biomarker measurement protocols are needed.

## Figures and Tables

**Table 1:** Demographic, clinical, and biochemical characteristics of pooled COVID-19 patient population and stratified as survivors and non-survivors.

	Total Cohort (*n* = 549)	Survivors (*n* = 443)	Non-Survivors (*n* = 106)	*p* Value
Age	72 (59–81)	75 (58–80)	77.5 (68–86)	<0.001
Sex (male), *n* (%)	261 (47.5)	210 (47.4)	51 (48.1)	
Total dose of remdesivir	600 (600–600)	600 (600–600)	600 (600–600)	0.264
Length of hospitalization, days	10.1 (5–13)	8 (5–12)	12 (7–20)	<0.001
Days of treatment	1 (0–2)	1 (0–2)	0 (0–2)	0.258
Intubation, *n* (%)	34 (6.2)	2 (0.5)	32 (30.2)	<0.001
COVID-19 vaccination (before hospitalization) *n* (%)	223 (40.6)	183 (41.3)	40 (37.7)	0.664
**Comorbidity,** ***n*** **(%)**				
Pulmonary disease	59 (16.2)	56 (12.6)	13 (12.3)	0.315
Hypertension	360 (65.6)	283 (63.9)	77 (72.6)	0.081
CVDs	193 (35.2)	140 (31.6)	52 (49.1)	0.006
Type 2 diabetes mellitus	160 (29.1)	131 (29.6)	29 (27.4)	0.504
Obesity	65 (11.8)	52 (11.7)	13 (12.3)	0.441
Carcinoma	37 (6.7)	26 (5.9)	11 (10.4)	0.087
**COVID-19 symptoms,** ***n*** **(%)**				
Fever	342 (62.3)	285 (64.3)	57 (53.8)	0.026
Headache	55 (10)	50 (11.3)	5 (4.7)	0.246
Nausea	40 (7.3)	35 (7.9)	5 (4.7)	0.701
Dyspnea	268 (48.8)	207 (46.7)	61 (57.5)	0.507
Irritability/confusion	21 (3.8)	13 (2.9)	8 (7.5)	0.069
Diarrhea	25 (4.6)	21 (4.7)	4 (3.8)	0.831
Cough	355 (64.7)	299 (67.5)	56 (52.8)	<0.001
Pharyngalgia	32 (5.8)	28 (6.3)	4 (3.8)	0.239
Arthralgia	28 (5.1)	26 (5.9)	2 (1.9)	0.051
Thoracic pain	16 (2.9)	13 (2.9)	3 (2.8)	0.638
Abdominal pain	11 (2)	10 (2.3)	1 (0.9)	0.941
**Biochemical markers**				
**Baseline (at admission and before remdesivir treatment)**				
Troponin (pg/mL)	14.1 (5.7–30.1), n = 445	11.1 (4.3–22.3), n = 354	23 (14.3–53.7), n = 91	<0.001
Fibrinogen (mg/dL)	429 (371–485), n = 337	430 (373–485), n = 275	410 (356.3–471), n = 62	0.120
D-dimers (ng/mL)	459 (289–738.5), n = 397	412 (267–713), n = 323	560 (337–853.8), n = 55	0.004
DFR	0.101 (0.062–0.181), n = 290	0.096 (0.058–0.172), n = 244	0.147 (0.085–0.225), n = 46	<0.001
**After Remdesivir Treatment**				
Troponin (pg/mL)	11.5 (5.2–25.5), n = 497	9.6 (4.7–21.1), n = 404	32.2 (13.7–66.4), n = 93	0.201
Fibrinogen (mg/dL)	429 (293.8–430.3), n = 434	367 (304–429), n = 359	344 (271–437), n = 75	<0.001
D-dimers (ng/mL)	429.5 (242.3–714.3), n = 422	392 (223.5–618.8), n = 350	771 (524.3–1369), n = 72	0.003
DFR	0.114 (0.063–0.0193), n = 360	0.101 (0.058–0.171), n = 305	0.198 (0.128–0.516), n = 55	<0.001

Data is shown as median and interquartile range or as n (%). Mann–Whitney test was applied for continuous variables. Chi-square was performed for categorical variables. DFR = D-dimer/fibrinogen ratio. *p* values derive from comparisons between the two groups (survivors vs. non-survivors).

**Table 2:** ROC curve analysis and cut-off values of troponin, D-dimers, and DFR to predict mortality at baseline and after remdesivir treatment.

	Area Under the Curve	Std. Error	*p* Value	95% CI	Cut-Off Value	Sensitivity (%)	Specificity (%)
Troponin baseline	0.715	0.029	<0 .001	0.659–0.772	12.7 pg/mL	80.2	53.7
Troponin after remdesivir treatment	0.759	0.028	<0.001	0.703–0.814	31.1 pg/mL	53.8	86.1
D-dimers baseline	0.606	0.035	0.002	0.539–0.674	479 ng/mL	68.9	57.9
D-dimers after remdesivir treatment	0.773	0.029	<0.001	0.716–0.830	524 ng/mL	76.4	66
DFR baseline	0.638	0.042	0.001	0.556–0.719	0.124	63	63.1
DFR after remdesivir treatment	0.765	0.033	<0.001	0.701–0.828	0.101	89.1	51.3

Std. Error = standard error; DFR = D-dimer/fibrinogen ratio; 95% CI = 95% confidence interval.

**Table 3:** Cox regression model evaluating troponin levels for death risk in COVID-19 patients at admission.

	HR (≥12.7 vs. <12.6 pg/mL)	95% CI	*p* Value
Model 1	2.686	1.580–4.568	<0.001
Model 2	1.808	0.936–3.491	0.078
Model 3	2.374	1.343–4.197	0.003
Model 4	1.734	0.885–3.399	0.083

Model 1 is the unadjusted troponin model. Model 2 includes troponin with covariates: age and sex. Model 3 includes troponin with covariates: pulmonary disease, CVDs, hypertension, carcinoma, obesity, and type 2 diabetes mellitus. Model 4 includes combination of covariates of model 2 and model 3. HR = hazard ratio; 95% CI = 95% confidence interval.

**Table 4:** Cox regression model evaluating troponin levels for death risk in COVID-19 patients after remdesivir treatment.

	HR (≥31.1 vs. <31.0 pg/mL)	95% CI	*p* Value
Model 1	2.662	1.762–4.020	<0.001
Model 2	2.063	1.275–3.336	0.003
Model 3	2.419	1.556–3.761	<0.001
Model 4	2.010	1.219–3.316	0.006

Model 1 is the unadjusted troponin model. Model 2 includes troponin with covariates: age and sex. Model 3 includes troponin with covariates: pulmonary disease, CVDs, hypertension, carcinoma, obesity, and type 2 diabetes mellitus. Model 4 includes combination of covariates of model 2 and model 3. HR = hazard ratio; 95% CI = 95% confidence interval.

**Table 5:** Cox regression model evaluating D-dimers for death risk in COVID-19 patients at admission.

	HR (≥ 479 vs. <478 ng/mL)	95% CI	*p* Value
Model 1	1.862	1.127–3.076	0.015
Model 2	1.583	0.946–2.648	0.080
Model 3	1.618	0.966–2.711	0.067
Model 4	1.477	0.877–2.489	0.143

Model 1 is the unadjusted D-dimers model. Model 2 includes D-dimers with covariates age and sex. Model 3 includes D-dimers with covariates: pulmonary disease, CVDs, hypertension, carcinoma, obesity, and type 2 diabetes mellitus. Model 4 includes combination of covariates of model 2 and model 3. HR = hazard ratio; 95% CI = 95% confidence interval.

**Table 6:** Cox regression model evaluating D-dimers for death risk in COVID-19 patients after remdesivir treatment.

	HR (≥524 vs. <523 ng/mL)	95% CI	*p* Value
Model 1	2.609	1.502–4.532	<0.001
Model 2	2.260	1.297–3.939	0.004
Model 3	2.429	1.382–4.269	0.002
Model 4	2.207	1.254–3.882	0.006

Model 1 is the unadjusted D-dimers model. Model 2 includes D-dimers with covariates: age and sex. Model 3 includes D-dimers with covariates: pulmonary disease, CVDs, hypertension, carcinoma, obesity, and type 2 diabetes mellitus. Model 4 includes a combination of covariates of model 2 and model 3. HR = hazard ratio; 95% CI = 95% confidence interval.

**Table 7:** Cox regression model evaluating DFR for death risk in COVID-19 patients at admission.

	HR (≥124.9 vs. <124.8)	95% CI	*p* Value
Model 1	1.655	0.892–3.072	0.110
Model 2	1.207	0.633–2.304	0.568
Model 3	1.469	0.777–2.778	0.236
Model 4	1.226	0.637–2.358	0.541

Model 1 is the unadjusted DFR model. Model 2 includes DFR with covariates: age and sex. Model 3 includes DFR with covariates: pulmonary disease, CVDs, hypertension, carcinoma, obesity, and type 2 diabetes mellitus. Model 4 includes a combination of covariates of model 2 and model 3. HR = hazard ratio; 95% CI = 95% confidence interval.

**Table 8:** Cox regression model evaluating DFR for death risk in COVID-19 patients after remdesivir treatment.

	HR (≥101.8 vs. <101.7)	95% CI	*p* Value
Model 1	4.616	1.974–10.798	<0.001
Model 2	3.797	1.591–9.063	<0.001
Model 3	4.252	1.790–10.097	0.001
Model 4	3.816	1.567–9.291	0.003

Model 1 is the unadjusted DFR model. Model 2 includes DFR with covariates: age and sex. Model 3 includes DFR with covariates: pulmonary disease, CVDs, hypertension, carcinoma, obesity, and type 2 diabetes mellitus. Model 4 includes a combination of covariates of model 2 and model 3. HR = hazard ratio; 95% CI = 95% confidence interval.

## Data Availability

The original contributions presented in this study are included in the article. Further inquiries can be directed at the corresponding author.

## Abbreviations

The following abbreviations are used in this manuscript:

COVID-19	Coronavirus disease
ESCMID	European Society of Clinical Microbiology and Infectious Diseases
VTE	Venous thromboembolism
DFR	D-dimers/fibrinogen ratio
ICU	Intensive care unit
IQR	Interquartile range
ROC	Receiver operator characteristics
CVDs	Cardiovascular disease
TF	Tissue factor
IL-6	Interleukin-6
CRP	C-reactive protein
aPTT	Activated partial thromboplastin time
PT	Prothrombin time
